# Thyroid function and thyroid homeostasis parameters are associated with increased urinary albumin excretion in euthyroid individuals over 60 years old from NHANES

**DOI:** 10.3389/fendo.2023.1285249

**Published:** 2024-01-08

**Authors:** Xue Liu, Yuchen Li, Yuwei Chai, Yuhao Zhang, Li Zhang, Haiqing Zhang

**Affiliations:** ^1^ Department of Endocrinology, Shandong Provincial Hospital, Shandong University, Jinan, Shandong, China; ^2^ Key Laboratory of Endocrine Glucose and Lipids Metabolism and Brain Aging, Ministry of Education, Department of Endocrinology, Shandong Provincial Hospital Affiliated to Shandong First Medical University, Jinan, Shandong, China; ^3^ Department of Urology, The Affiliated Hospital of Qingdao University, Qingdao, China; ^4^ Department of Vascular Surgery, Shandong Provincial Hospital Affiliated to Shandong First Medical University, Jinan, Shandong, China; ^5^ Shandong Clinical Medical Center of Endocrinology and Metabolism, Jinan, China; ^6^ Institute of Endocrinology and Metabolism, Shandong Academy of Clinical Medicine, Jinan, China

**Keywords:** thyroid function, thyroid homeostasis parameters, albuminuria, National Health and Nutrition Examination Survey (NHANES), cross-sectional study

## Abstract

**Introduction:**

The relationship between thyroid function/homeostasis parameters and renal function has been extensively studied. However, the relationship between thyroid function and thyroid homeostasis parameters with albuminuria among elderly individuals remains unclear.

**Methods:**

The population was divided into an albuminuria group and a non-albuminuria group for baseline characteristic difference analysis. Multivariable logistic regression was used to test the association between thyroid function, and thyroid homeostasis parameters and albuminuria. The nonlinear relationship was explored with restricted cubic splines. Meanwhile, we investigated whether the relationship also existed in the diabetes and hypertension subgroups. Receiver operating characteristic (ROC) curves were used to assess the effectiveness of the indices.

**Results:**

FT4 and TFQI_FT4_ were positively correlated with albuminuria (OR = 1.12; 95% CI = 1.02–1.23, *p* = 0.02; OR = 1.79; 95% CI = 1.08–2.99, *p* = 0.03), and FT3/FT4 was negatively correlated with albuminuria (OR = 0.03; 95% CI = 0.00–0.26, *p* = 0.003). Additionally, the nonlinear relationship between FT3/FT4 as well as TSHI and albuminuria was approximately U-shaped. Similar results were observed in the hypertension subgroup but not in the diabetes subgroup. There was a U-shaped nonlinear relationship between FT3 and albuminuria in the diabetes group. In addition, FT3/FT4 performed better than TFQI, TT4RI, and TSHI in ROC analyses for albuminuria prediction.

**Conclusion:**

FT4, TFQI_FT4_, and a low FT3/FT4 ratio were risk factors for albuminuria in euthyroid individuals over 60 years old. However, FT3 was more associated with albuminuria in the diabetes subgroup. TSH was not associated with albuminuria in any analysis. In our study, we attempted to provide more reasonable thyroid parameters and basis for evaluating patients with underlying albuminuria. FT3/FT4 may be used as a helpful indicator to predict albuminuria and provide novel ideas for the evaluation and treatment of albuminuria.

## 1 Introduction

Thyroid function is related to energy metabolism and protein synthesis and is evaluated with serum free triiodothyronine (FT3), free thyroxine (FT4), and thyroid-stimulating hormone (TSH) levels. Thyroid hormones promote tissue growth, maturation, and differentiation (1) and are controlled by the hypothalamus–pituitary–thyroid (HPT) axis, which involves stimulation of thyroxine (T4) production by TSH from the pituitary (2). Thyroid homeostatic parameters, when combined with thyroid hormones and the feedback of the HPT axis, can offer a novel interpretation of the current thyroid status. The initial assessment of central sensitivity to thyroid hormones is conducted using TSHI (TSH index) and TT4RI and TT3RI (thyrotrophic T4 and T3 resistance indices) (3, 4). The higher the values, the lower the central sensitivity to thyroid hormones. The thyroid feedback quantile-based index (TFQI_FT4_, TFQI_FT3_) proposed by Laclaustra et al. was thought to be more stable than TSHI and TT4RI (5). The negative values indicated higher sensitivity to FT4 in the pituitary, positive values indicated less sensitivity, and the value of 0 indicated a normal sensitivity. In thyroidal and peripheral tissues, FT4 is converted to the active free triiodothyronine (FT3) hormone through a process that can be assessed using the FT3:FT4 ratio (5, 6).

Albuminuria, alternatively referred to as elevated urinary albumin excretion (UAE), is commonly defined as a urinary albumin-to-creatinine ratio (UACR) equal to or greater than 30 mg/g (7–9). Microalbuminuria (30–300 mg/g) has been documented to be present in 5%–19% of the overall population, with prevalence rising to 23% in individuals with hypertension and escalating further to 40% in those with diabetes (10). During the initial phases of glomerular disease, even when routine urine tests show negative results for urine protein, the levels of urinary microalbumin can fluctuate. This phenomenon is frequently regarded as the most sensitive and dependable diagnostic marker for the early identification of chronic kidney disease (CKD) in cases where the estimated glomerular filtration rate (eGFR) is within the normal range. Furthermore, it is linked to unfavorable health outcomes (11, 12). In addition, UAE has also been shown to be an independent predictor of CKD progression and cardiovascular risk (7, 13).

Chen et al. reported that UACR levels were negatively associated with FT3 and triiodothyronine (T3) in patients with type 2 diabetes (14). Yang et al. demonstrated a positive relationship between eGFR calculated using the CKD Epidemiology Collaboration (CKD-EPI) equation and the FT3/FT4 ratio, while an inverse correlation was observed with PTFQI_FT4_. Furthermore, TSHI, TT4RI, and TT3RI displayed a negative correlation with renal function, as defined by a decline in eGFR (15). However, no studies have confirmed the relationship between thyroid hormone and thyroid homeostasis parameters and UAE in the general population.

With aging, there may be changes to renal tissues resulting in an increase in the permeability of the glomerular filtration membrane. As a result, albuminuria is more common in elderly individuals. Therefore, identifying factors that influence albuminuria in the elderly population is of particular clinical importance. In this study, we investigated the relationship between thyroid function, thyroid homeostatic parameters, and increased UAE in individuals aged 60 and above using data from the National Health and Nutrition Examination Survey (NHANES) 2007–2012 to provide a more specific thyroid management strategy for albuminuria patients.

## 2 Materials and methods

### 2.1 Study population

**Figure 1:**
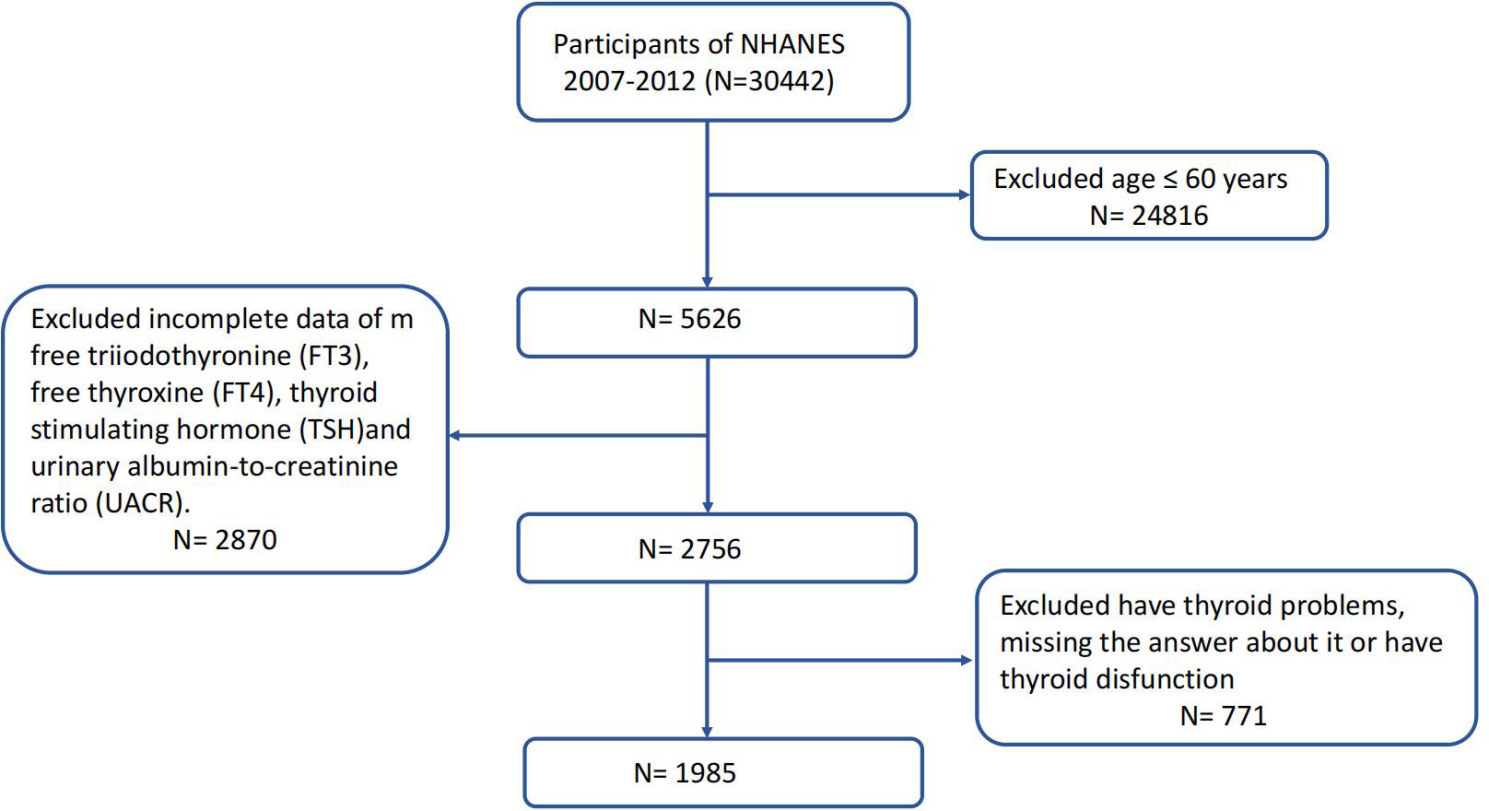
Study flowchart of the National Health and Nutrition Examination Survey (NHANES), 2007–2012.

NHANES is a series of research initiatives aimed at evaluating the health and nutritional wellbeing of both adults and children across the United States. The National Center for Health Statistics (NCHS) is a component of the Centers for Disease Control and Prevention (CDC) and is tasked with generating crucial statistics pertaining to health and overall wellbeing at a national level. NHANES studies receive approval from the Research Ethics Review Board of the US National Center for Health Statistics, and all participants provide written informed consent. Comprehensive information is available through the NHANES website (www.cdc.gov/nchs/nhanes/index.htm). In our investigation, we amalgamated data from three NHANES cycles (2007–2008, 2009–2010, and 2011–2012). After the exclusion of participants aged <60, those who had thyroid problems or thyroid dysfunction, and those with missing data about FT3, FT4, TSH, and UACR, our final analysis included 1,985 eligible participants who were representative of the U.S. population aged 60 years and older. **Figure 1** depicts the full data integration process.

### 2.2 Assessment of thyroid function and thyroid homeostasis parameters

Serum FT3 and FT4 levels were assessed using a competitive binding immunoenzymatic assay and a two-step enzyme immunoassay, respectively. TSH levels were quantified using the Access HYPERsensitive hTSH Assay, employing a two-site immunoenzymatic (“sandwich”) technique. The normal references for thyroid hormone levels in NHANES 2007–2012 were as follows: serum FT4 level of 7.74–20.64 pmol/L, serum FT3 level of 2.5-3.9 ng/dL, and TSH level of 0.34–5.60 mIU/L (5).

The process by which FT4 is converted to active FT3 can be assessed by the FT3/FT4 ratio, i.e., FT3 (pmol/L)/FT4 (pmol/L) (16). Central indices of thyroid hormone sensitivity were computed using the formulas presented below: TFQI was derived by applying the population empirical cumulative distribution function (cdf) to hormone concentration (5), representing the thyroid feedback quantile-based index; TFQI_FT4_ was determined as cdf FT4 − (1 − cdf TSH); TFQI_FT3_ was calculated as cdf FT3 − (1 − cdf TSH); the TFQI values range between −1 and 1, where negative and positive values denote good and poor sensitivity to FT4 or FT3, respectively (17); TT4RI was calculated as FT4 (pmol/L) * TSH (mIU/L) (4); TT3RI was calculated as FT3 (pmol/L) * TSH (mIU/L); TSHI was calculated as ln TSH (mIU/L) + 0.1345 * FT4 (pmol/L) (3). Higher values of TSHI, TT4RI, and TT3RI indicated reduced central sensitivity to thyroid hormones (18).

### 2.3 Assessment of albuminuria

Urinary albumin and creatinine levels were measured from a single spot urine sample utilizing solid-phase fluorescence immunoassay and a modified Jaffe kinetic method (12). The UACR was computed by dividing the concentration of urinary albumin (mg) by the concentration of urinary creatinine (g). Typically, albuminuria is defined as a UACR equal to or exceeding 30 mg/g (7–9).

### 2.4 Covariates

Details pertaining to individuals’ sociodemographic factors, health behaviors, and health-related status were gathered through interviews based on questionnaires and mobile examination centers (MECs).

Sociodemographic factors included age (years), sex (male and female), race (Mexican American, Non-Hispanic black, Non-Hispanic white, Other Hispanic, and Other race), and education levels (less than 9th grade, 9–11th grade, and more than high school). Health behaviors included smoking (never smoker, former smoker, and now smoker). Individuals who had not smoked 100 cigarettes in their entire lives were categorized as never smokers. Those who had smoked 100 cigarettes at some point in their lives were designated former smokers if they responded “No” to the question “Do you smoke now?” and as current smokers if they responded “Yes” (19). Health-related status included body mass index (BMI) (kg/m^2^), alanine aminotransferase (ALT) (U/L), aspartate aminotransferase (AST) (U/L), uric acid (µmol/L), triglycerides (mmol/L), total cholesterol (mmol/L), urine iodine (µg/L), estimated glomerular filtration rate (eGFR) (mL/min/1.73 m^2^), diabetes (yes or no), and hypertension (yes or no). BMI was calculated as weight in kilograms divided by height in meters squared. ALT and AST were incorporated because hepatic histological abnormalities exhibit a notable association with abnormal albuminuria (20). eGFR was determined utilizing the CKD-EPI equation, incorporating the calibrated creatinine level (21). Diabetes was defined as having a glycohemoglobin level of ≥6.5%, being on diabetes medication or insulin treatment, or self-reporting a diagnosis of diabetes (22). Hypertension was defined as the utilization of antihypertensive medications, a clinical diagnosis of hypertension, or three successive measurements of systolic blood pressure equal to or greater than 140 mmHg or diastolic blood pressure equal to or greater than 90 mmHg (23). A detailed description of the variables used in this research is available at https://www.cdc.gov/nchs/nhanes/.

### 2.5 Statistical analysis

The choice of analysis weights was according to the guidelines outlined in the NHANES database instructions. Weighted mean and standard error (SE) were used to denote baseline characteristics for continuous variables, while weighted proportions were employed for categorical variables. Weighted multivariate logistic regression models were employed to calculate OR and its corresponding 95% confidence interval in relation to albuminuria. In Model 1, no variable was adjusted for. In Model 2, sociodemographic data (age, sex, education level, and race), smoking, and BMI were adjusted for, and AST, ALT, triglyceride, total cholesterol, uric acid, urine iodine, eGFR, diabetes, and hypertension were further adjusted in Model 3. The nonlinear relationship between thyroid function and thyroid homeostasis parameters and albuminuria was characterized by restricted cubic splines after adjustments for covariates. Meanwhile, we explored baseline characteristics and the linear and nonlinear relationship between thyroid function and thyroid homeostasis parameters with albuminuria in the diabetes and hypertension subgroups. To assess the effectiveness of the indices, we analyzed the receiver operating characteristic (ROC) curves, depicting sensitivity versus 1 − specificity, and derived the cutoff points based on the ROC outcomes.

We describe the proportion of missing covariates (**Supplementary Table 1**). For missing covariates, we created imputed datasets with chained equations. The “mice” package was used for multiple imputations on samples with missing covariate data. We conducted sensitivity analysis of the results after imputations of the covariates that needed to be filled (**Supplementary Table 2**).

*p* < 0.05 was considered statistically significant. All NHANES analyses accounted for the complex survey design, with the weighted analysis being conducted by the survey package in R (4.3.1) software.

## 3 Results

### 3.1 Participant characteristics

All participants were categorized into two groups based on their UACR results: UACR <3 0 mg/g (*n* = 1,526) and UACR ≥30 mg/g (*n* = 459). There was a significant difference in age, race, education levels, uric acid, eGFR, DM or not, hypertension or not, FT3, FT4, FT3/FT4, TFQI_FT4_, TT4RI, and TSHI (*p* < 0.05) (see **Table 1**).

**Table 1:** Comparison of characteristics based on albuminuria or not.

Variable	Total (*n* = 1,985)	UACR < 30 mg/g (*n* = 1,526)	UACR ≥ 30 mg/g (*n* = 459)	*p*-value
Age (years)	70.30 ± 0.21	69.63 ± 0.26	73.21 ± 0.44	<0.0001*
Sex (***N***, %)				0.42
Female	899 (45.29)	707 (49.97)	192 (47.18)	
Male	1,086 (54.71)	819 (50.03)	267 (52.82)	
Race (***N***, %)				0.003*
Mexican American	212 (10.68)	150 (3.01)	62 (6.39)	
Non-Hispanic black	414 (20.86)	309 (8.62)	105 (11.28)	
Non-Hispanic white	1,043 (52.54)	821 (79.78)	222 (71.13)	
Other Hispanic	203 (10.23)	165 (3.72)	38 (4.53)	
Other race	113 (5.69)	87 (4.87)	32 (6.67)	
Education levels (***N***, %)				<0.0001*
Less than 9th grade	395 (19.92)	274 (9.98)	121 (18.99)	
9–11th grade	327 (16.49)	237 (13.96)	90 (19.68)	
More than high school	1,261 (63.59)	1,014 (76.06)	247 (61.33)	
Smoking (***N***, %)				0.16
Never smoker	928 (46.80)	735 (46.68)	193 (39.44)	
Former smoker	813 (41.00)	618 (43.55)	195 (46.64)	
Current smoker	242 (12.20)	171 (9.77)	71 (13.91)	
BMI (kg/m^2^)	28.56 ± 0.21	28.56 ± 0.23	28.57 ± 0.44	0.98
ALT (U/L)	23.22 ± 0.55	23.22 ± 0.53	23.24 ± 1.73	0.99
AST (U/L)	25.77 ± 0.37	25.71 ± 0.40	26.04 ± 0.99	0.75
Uric acid (μmol/L)	340.34 ± 2.86	337.69 ± 3.23	351.68 ± 5.86	0.03*
Triglyceride (mmol/L)	1.44 ± 0.02	1.41 ± 0.03	1.54 ± 0.06	0.07
Total cholesterol (mmol/L)	5.09 ± 0.05	5.13 ± 0.05	4.93 ± 0.09	0.06
Urine iodine (μg/L)	382.08 ± 49.06	365.12 ± 52.23	454.72 ± 116.60	0.48
eGFR (mL/min/1.73 m^2^)	73.46 ± 0.63	75.12 ± 0.68	66.37 ± 1.45	<0.0001*
Diabetes or not				<0.0001*
Yes	592 (29.82)	368 ± 19.58	224 ± 41.00	
No	1,393 (70.18)	1,158 ± 80.42	235 ± 59.00	
Hypertension or not				<0.0001*
Yes	1,401 (70.58)	1,028 ± 64.13	373 ± 81.64	
No	584 (29.42)	498 ± 35.87	86 ± 18.36	
FT3 (pg/mL)	2.97 ± 0.01	2.99 ± 0.01	2.90 ± 0.02	<0.001*
FT4 (pmol/L)	10.77 ± 0.05	10.69 ± 0.06	11.14 ± 0.11	<0.001*
TSH (mIU/L)	1.99 ± 0.04	1.97 ± 0.04	2.11 ± 0.08	0.11
FT3/FT4	0.43 ± 0.00	0.44 ± 0.00	0.41 ± 0.00	<0.0001*
TFQI_FT4_	0.09 ± 0.02	0.07 ± 0.02	0.17 ± 0.03	0.002*
TFQI_FT3_	0.02 ± 0.02	0.03 ± 0.02	−0.02 ± 0.03	0.09
TT4RI	21.33 ± 0.41	20.89 ± 0.44	23.22 ± 0.89	0.02*
TT3RI	9.10 ± 0.16	9.05 ± 0.19	9.35 ± 0.33	0.43
TSHI	2.02 ± 0.02	2.00 ± 0.02	2.11 ± 0.04	0.02*

Data were presented as mean ± SD or median (interquartile ranges) for continuous variables, and numbers (proportions) for categorical variables.

BMI, body mass index; ALT, alanine aminotransferase; AST, aspartate aminotransferase; eGFR, estimated glomerular filtration rate; FT3, free triiodothyronine; FT4, free thyroxine; TSH, thyroid-stimulating hormone; FT3/FT4, FT3/FT4 ratio; TFQI_FT4_, TFQI_FT3_, thyroid feedback quantile-based index; TT4RI, thyrotrophic T4 resistance index; TT3RI, thyrotrophic T3 resistance index; TSHI, TSH index.

*p < 0.05.

**Figure 2:**
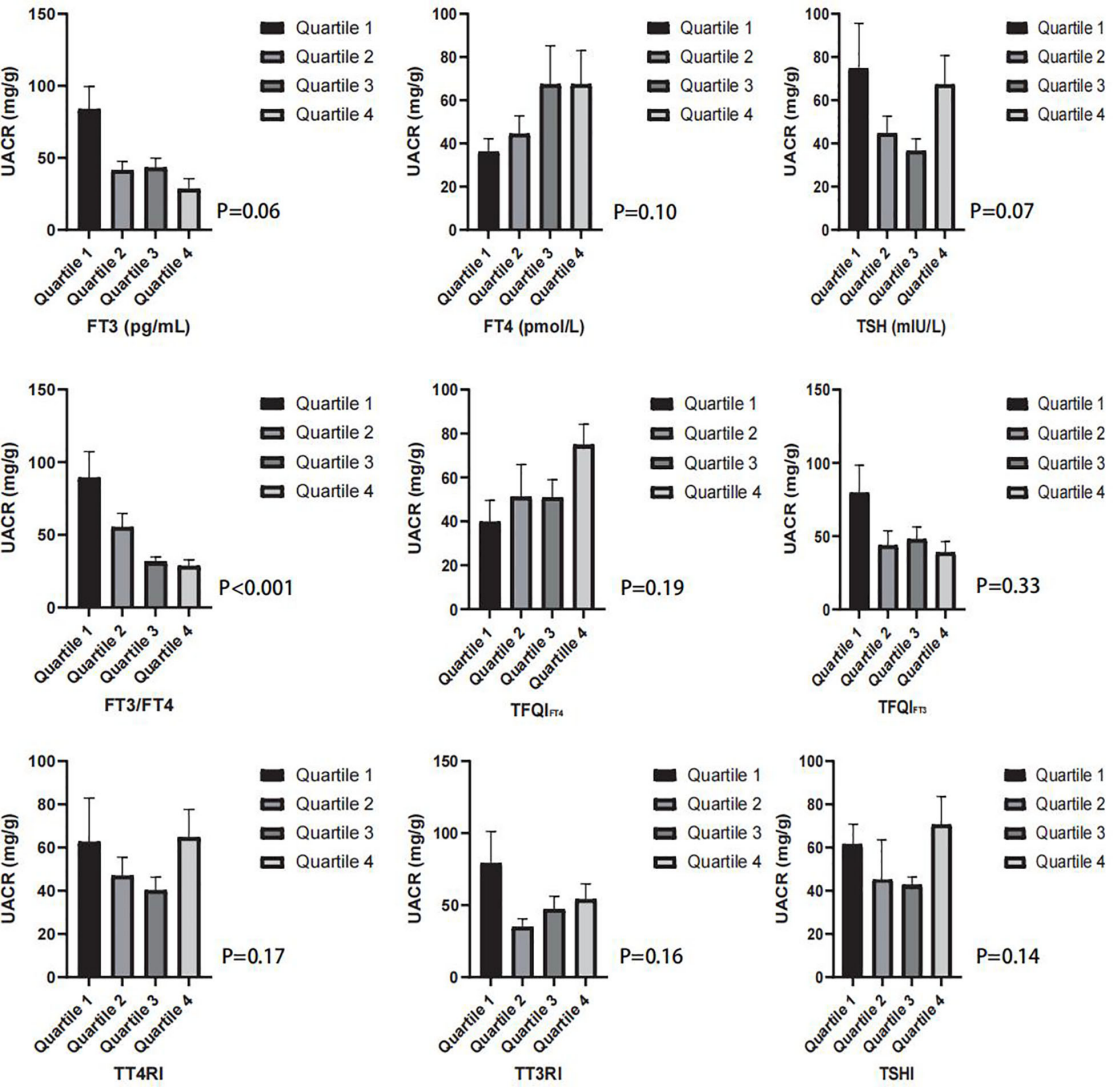
The box graph shows the mean UACR (mg/g) in the quartiles of FT3/FT4, TFQIFT4, TFQIFT3, TT4RI, TT3RI, and TSHI groups.

There was also a significant difference in age, race, education levels, eGFR, FT3/FT4, and TFQI_FT4_ between the albuminuria and non-albuminuria groups in both the diabetes and hypertension subgroups (see **Table 2**). With the division of FT3, FT4, TSH, FT3/FT4, TFQI_FT4_, TFQI_FT3_, TT4RI, TT3RI, and TSHI into quartiles, there was only a significant difference in UACR with the consideration of FT3/FT4 (see **Figure 2**).

**Table 2:** Comparison of characteristics between the two groups of patients with diabetes and hypertension.

Variable	Diabetes			Hypertension		
	UACR < 30 mg/g (*n* = 368)	UACR ≥ 30 mg/g (*n* = 224)	*p*-value	UACR < 30 mg/g (*n* = 1,028)	UACR ≥ 30 mg/g (*n* = 373)	*p*-value
Age (years)	69.63 ± 0.26	73.21 ± 0.44	<0.0001*	70.11 ± 0.33	73.13 ± 0.51	<0.0001*
Sex (*N*, %)			0.42			0.55
Female	707 (49.97)	192 (47.18)		501 (50.71)	161 (48.19)	
Male	819 (50.03)	267 (52.82)		527 (49.29)	212 (51.81)	
Race (***N***, %)			0.003*			0.03*
Mexican American	150 (3.01)	62 (6.39)		103 (3.34)	48 (6.62)	
Non-Hispanic black	309 (8.62)	105 (11.28)		237 (10.71)	91 (12.18)	
Non-Hispanic white	821 (79.78)	222 (71.13)		530 (76.67)	173 (69.22)	
Other Hispanic	165 (3.72)	38 (4.53)		106 (3.84)	33 (5.18)	
Other race	81 (4.87)	32 (6.67)		52 (5.44)	28 (6.81)	
Education levels (*N*, %)			<0.0001*			0.002*
Less than 9th grade	274 (9.98)	121 (18.99)		187 (11.18)	93 (19.09)	
9–11th grade	237 (13.96)	90 (19.68)		163 (13.94)	75 (20.32)	
More than high school	1,014 (76.06)	247 (61.33)		678 (74.88)	205 (60.59)	
Smoking (*N*, %)			0.16			0.10
Never smoker	735 (46.68)	193 (39.44)		511 (49.34)	162 (40.87)	
Former smoker	618 (43.55)	195 (46.64)		415 (43.80)	160 (47.46)	
Current smoker	171 (9.77)	71 (13.91)		101 (6.86)	51 (11.66)	
BMI (kg/m^2^)	28.56 ± 0.23	28.57 ± 0.44	0.98	29.16 ± 0.29	29.05 ± 0.45	0.84
ALT (U/L)	23.22 ± 0.53	23.24 ± 1.73	0.99	23.96 ± 0.76	22.69 ± 1.40	0.40
AST (U/L)	25.71 ± 0.40	26.04 ± 0.99	0.75	26.27 ± 0.53	25.35 ± 0.98	0.37
Uric acid (μmol/L)	337.69 ± 3.23	351.68 ± 5.86	0.03*	346.38 ± 4.54	351.78 ± 6.65	0.50
Triglyceride (mmol/L)	1.41 ± 0.03	1.54 ± 0.06	0.07	1.46 ± 0.04	1.53 ± 0.06	0.38
Total cholesterol (mmol/L)	5.13 ± 0.05	4.93 ± 0.09	0.06	5.03 ± 0.06	4.99 ± 0.09	0.76
Urine iodine (μg/L)	365.12 ± 52.23	454.72 ± 116.60	0.48	434.59 ± 81.00	485.02 ± 141.33	0.75
eGFR (mL/min/1.73 m^2^)	75.12 ± 0.68	66.37 ± 1.45	<0.0001*	73.23 ± 0.84	65.61 ± 1.68	<0.001*
**FT3 (pg/mL)**	2.99 ± 0.01	2.90 ± 0.02	<0.001*	2.98 ± 0.02	2.91 ± 0.03	0.04*
**FT4 (pmol/L)**	10.69 ± 0.06	11.14 ± 0.11	<0.001*	10.72 ± 0.06	11.17 ± 0.12	<0.001*
**TSH (mIU/L)**	1.97 ± 0.04	2.11 ± 0.08	0.11	2.00 ± 0.05	2.11 ± 0.09	0.25
**FT3/FT4**	0.44 ± 0.00	0.41 ± 0.00	<0.0001*	0.44 ± 0.00	0.41 ± 0.01	<0.001*
**TFQI_FT4_**	0.07 ± 0.02	0.17 ± 0.03	0.002*	0.08 ± 0.02	0.17 ± 0.03	0.02*
**TFQI_FT3_**	0.03 ± 0.02	−0.02 ± 0.03	0.09	0.04 ± 0.02	−0.01 ± 0.03	0.16
**TT4RI**	20.89 ± 0.44	23.22 ± 0.8	0.02*	21.24 ± 0.61	23.30 ± 0.99	0.07
**TT3RI**	9.05 ± 0.19	9.35 ± 0.33	0.43	9.14 ± 0.22	9.39 ± 0.36	0.53
**TSHI**	2.00 ± 0.02	2.11 ± 0.04	0.02*	2.01 ± 0.03	2.11 ± 0.05	0.06

Data were presented as mean ± SD or median (interquartile ranges) for continuous variables, and numbers (proportions) for categorical variables.

BMI, body mass index; ALT, alanine aminotransferase; AST, aspartate aminotransferase; eGFR, estimated glomerular filtration rate; FT3, free triiodothyronine; FT4, free thyroxine; TSH, thyroid-stimulating hormone; FT3/FT4, FT3/FT4 ratio; TFQI_FT4_, TFQI_FT3_, thyroid feedback quantile-based index; TT4RI, thyrotrophic T4 resistance index; TT3RI, thyrotrophic T3 resistance index; TSHI, TSH index.

*p < 0.05.

### 3.2 Correlations of thyroid function with albuminuria

We conducted weighted multivariate logistic regression models to explore the association between thyroid function and albuminuria (see **Table 3**). After adjusting for age, sex, education level, race, smoking status, BMI, ALT, AST, triglycerides, total cholesterol, uric acid, eGFR, urine iodine, DM, and hypertension (Model 3), we found that FT4 was positively correlated with albuminuria (OR = 1.12; 95% CI = 1.02–1.23, *p* = 0.02). In the fully adjusted model, the results of multivariate logistic regression are shown in **Supplementary Tables 3–5**. There was no relationship between either FT3 or TSH and albuminuria (OR = 0.62, 95% CI = 0.34–1.11, *p* = 0.10; OR = 1.11, 95% CI = 0.90–1.36, *p* = 0.33). In restricted cubic splines, there was no nonlinear relationship between FT3, FT4, and TSH and albuminuria (*p* for all >0.05 or *p* for nonlinear >0.05).

**Table 3:** Association between thyroid function with albuminuria.

	Model 1		Model 2		Model 3	
	OR (95% CI)	*p*-value	OR (95% CI)	*p*-value	OR (95% CI)	*p*-value
FT3						
Continuous	0.39 (0.23,0.68)	0.001*	0.53 (0.30,0.95)	0.03*	0.62 (0.34,1.11)	0.10
Quartile 1	Ref	Ref	Ref	Ref	Ref	Ref
Quartile 2	0.56 (0.37,0.84)	0.01*	0.57 (0.39,0.84)	0.01*	0.63 (0.44,0.90)	0.01*
Quartile 3	0.57 (0.38,0.86)	0.01*	0.63 (0.40,1.00)	0.05	0.66 (0.41,1.07)	0.09
Quartile 4	0.50 (0.31,0.81)	0.01*	0.60 (0.36,1.00)	0.05	0.65 (0.39,1.08)	0.09
*p* for trend		0.005*		0.048*		0.096
FT4						
Continuous	1.19 (1.08,1.31)	<0.001*	1.14 (1.04,1.24)	0.004*	1.12 (1.02,1.23)	0.02*
Quartile 1	Ref	Ref	Ref	Ref	Ref	Ref
Quartile 2	1.10 (0.76,1.58)	0.61	1.07 (0.75,1.52)	0.70	1.08 (0.74,1.57)	0.68
Quartile 3	1.62 (1.10,2.41)	0.02*	1.52 (1.01,2.30)	0.04*	1.55 (0.99,2.42)	0.05
Quartile 4	2.05 (1.28,3.28)	0.004*	1.77 (1.15,2.71)	0.01*	1.73 (1.08,2.79)	0.03*
*p* for trend		0.003*		0.006*		0.012*
TSH						
Continuous	1.16 (0.97,1.38)	0.10	1.14 (0.95,1.38)	0.16	1.11 (0.90,1.36)	0.33
Quartile 1	Ref	Ref	Ref	Ref	Ref	Ref
Quartile 2	0.65 (0.41,1.04)	0.07	0.82 (0.46,1.44)	0.47	0.74 (0.46,1.19)	0.20
Quartile 3	0.79 (0.48,1.28)	0.33	0.88 (0.48,1.62)	0.67	0.86 (0.48,1.55)	0.61
Quartile 4	1.07 (0.65,1.75)	0.79	1.05 (0.59,1.90)	0.86	1.01 (0.56,1.79)	0.98
*p* for trend		0.560		0.561		0.816

Model 1: Non-adjusted.

Model 2: Adjusted for age, sex, education level, race, smoke, and BMI.

Model 3: Adjusted for age, sex, education level, race, smoke, BMI, ALT, AST, triglyceride, total cholesterol, uric acid, eGFR, urine iodine, DM, and hypertension.

FT3, free triiodothyronine; FT4, free thyroxine; TSH, thyroid-stimulating hormone.

*p < 0.05.

### 3.3 Correlations of thyroid homeostasis parameters with albuminuria

Likewise, we performed weighted multivariate logistic regression to investigate the potential relationship between thyroid homeostasis parameters and albuminuria (see **Table 4**). After adjusting for age, sex, education level, race, smoking status, BMI, ALT, AST, triglycerides, total cholesterol, uric acid, eGFR, urine iodine, DM, and hypertension (Model 3), we found that TFQI_FT4_ was positively correlated with albuminuria (OR = 1.79; 95% CI = 1.08–2.99, *p* = 0.03), and FT3/FT4 was negatively correlated with albuminuria (OR = 0.03; 95% CI = 0.00–0.26, *p* = 0.003). In the fully adjusted model, the results of multivariate logistic regression are shown in **Supplementary Tables 6–11**. When divided into quartiles, FT3/FT4 were still significantly associated with albuminuria. The ORs and 95% CIs from lowest to highest FT3/FT4 categories were 1.00 (reference), (OR = 0.79; 95% CI = 0.46–1.36, *p* = 0.38), (OR = 0.49; 95% CI = 0.30–0.80, *p* = 0.01), and (OR = 0.55; 95% CI = 0.33–0.92, *p* = 0.02), respectively, for albuminuria (*p* trend = 0.006) (see **Table 4**).

**Table 4:** Association between thyroid homeostasis parameters with albuminuria.

	Model 1		Model 2		Model 3	
	OR (95% CI)	*p*-value	OR (95% CI)	*p*-value	OR (95% CI)	*p*-value
FT3/FT4						
Continuous	0.00 (0.00,0.04)	<0.0001*	0.02 (0.00,0.15)	<0.001*	0.03 (0.00,0.26)	0.003*
Quartile 1	Ref	Ref	Ref	Ref	Ref	Ref
Quartile 2	0.69 (0.40,1.19)	0.17	0.74 (0.43,1.27)	0.27	0.79 (0.46,1.36)	0.38
Quartile 3	0.37 (0.22,0.61)	<0.001*	0.45 (0.28,0.72)	0.001*	0.49 (0.30,0.80)	0.01*
Quartile 4	0.43 (0.26,0.72)	0.002*	0.52 (0.32,0.85)	0.01*	0.55 (0.33,0.92)	0.02*
*p* for trend		<0.001*		0.002*		0.006*
TFQI_FT4_						
Continuous	2.14 (1.30,3.52)	0.003*	2.01 (1.26,3.22)	0.005*	1.79 (1.08,2.99)	0.03*
Quartile 1	Ref	Ref	Ref	Ref	Ref	Ref
Quartile 2	1.15 (0.75,1.77)	0.51	1.09 (0.70,1.71)	0.69	1.10 (0.68,1.78)	0.68
Quartile 3	1.44 (0.84,2.48)	0.18	1.25 (0.73,2.14)	0.41	1.19 (0.68,2.08)	0.54
Quartile 4	1.96 (1.21,3.16)	0.01*	1.80 (1.11,2.92)	0.02*	1.62 (0.94,2.79)	0.08
*p* for trend		0.005*		0.016*		0.077
TFQI_FT3_						
Continuous	0.68 (0.44,1.06)	0.09	0.81 (0.51,1.29)	0.37	0.84 (0.54,1.31)	0.42
Quartile 1	Ref	Ref	Ref	Ref	Ref	Ref
Quartile 2	0.95 (0.62,1.47)	0.82	1.10 (0.69,1.74)	0.68	1.09 (0.68,1.75)	0.71
Quartile 3	0.83 (0.48,1.42)	0.48	0.97 (0.57,1.67)	0.92	1.02 (0.59,1.76)	0.93
Quartile 4	0.64 (0.39,1.06)	0.08	0.79 (0.46,1.36)	0.38	0.78 (0.46,1.32)	0.34
*p* for trend		0.099		0.381		0.350
TT4RI						
Continuous	1.02 (1.00,1.04)	0.01*	1.02 (1.00,1.04)	0.03*	1.01 (1.00,1.03)	0.11
Quartile 1	Ref	Ref	Ref	Ref	Ref	Ref
Quartile 2	0.97 (0.64,1.48)	0.88	1.03 (0.65,1.64)	0.89	1.10 (0.70,1.76)	0.66
Quartile 3	0.87 (0.55,1.36)	0.53	0.88 (0.52,1.50)	0.64	0.86 (0.52,1.43)	0.54
Quartile 4	1.41 (0.90,2.22)	0.13	1.38 (0.87,2.20)	0.16	1.31 (0.78,2.21)	0.30
*p* for trend		0.166		0.244		0.471
TT3RI						
Continuous	1.01 (0.99,1.03)	0.19	1.02 (0.98,1.06)	0.40	1.01 (0.97,1.06)	0.57
Quartile 1	Ref	Ref	Ref	Ref	Ref	Ref
Quartile 2	0.63 (0.38,1.04)	0.07	0.61 (0.37,1.01)	0.05	0.63 (0.38,1.06)	0.08
Quartile 3	0.79 (0.47,1.31)	0.35	0.86 (0.50,1.48)	0.58	0.84 (0.48,1.48)	0.53
Quartile 4	0.96 (0.59,1.57)	0.87	0.95 (0.56,1.61)	0.84	0.88 (0.50,1.54)	0.64
*p* for trend		0.867		0.776		0.965
TSHI						
Continuous	1.50 (1.06,2.12)	0.02*	1.44 (1.02,2.03)	0.04*	1.32 (0.90,1.92)	0.15
Quartile 1	Ref	Ref	Ref	Ref	Ref	Ref
Quartile 2	0.96 (0.61,1.51)	0.85	1.07 (0.65,1.76)	0.79	1.11 (0.68,1.82)	0.67
Quartile 3	0.87 (0.55,1.37)	0.53	0.87 (0.52,1.45)	0.57	0.84 (0.51,1.38)	0.46
Quartile 4	1.56 (1.01,2.41)	0.04*	1.51 (0.97,2.36)	0.07	1.39 (0.84,2.29)	0.19
*p* for trend		0.055		0.118		0.330

Model 1: Non-adjusted.

Model 2: Adjusted for age, sex, education level, race, smoke, and BMI.

Model 3: Adjusted for age, sex, education level, race, smoke, BMI, ALT, AST, triglyceride, total cholesterol, uric acid, eGFR, urine iodine, DM, and hypertension.

FT3/FT4, FT3/FT4 ratio; TFQI_FT4_, TFQI_FT3_, thyroid feedback quantile-based index; TT4RI, thyrotrophic T4 resistance index; TT3RI, thyrotrophic T3 resistance index; TSHI, TSH index.

*p < 0.05.

**Figure 3:**
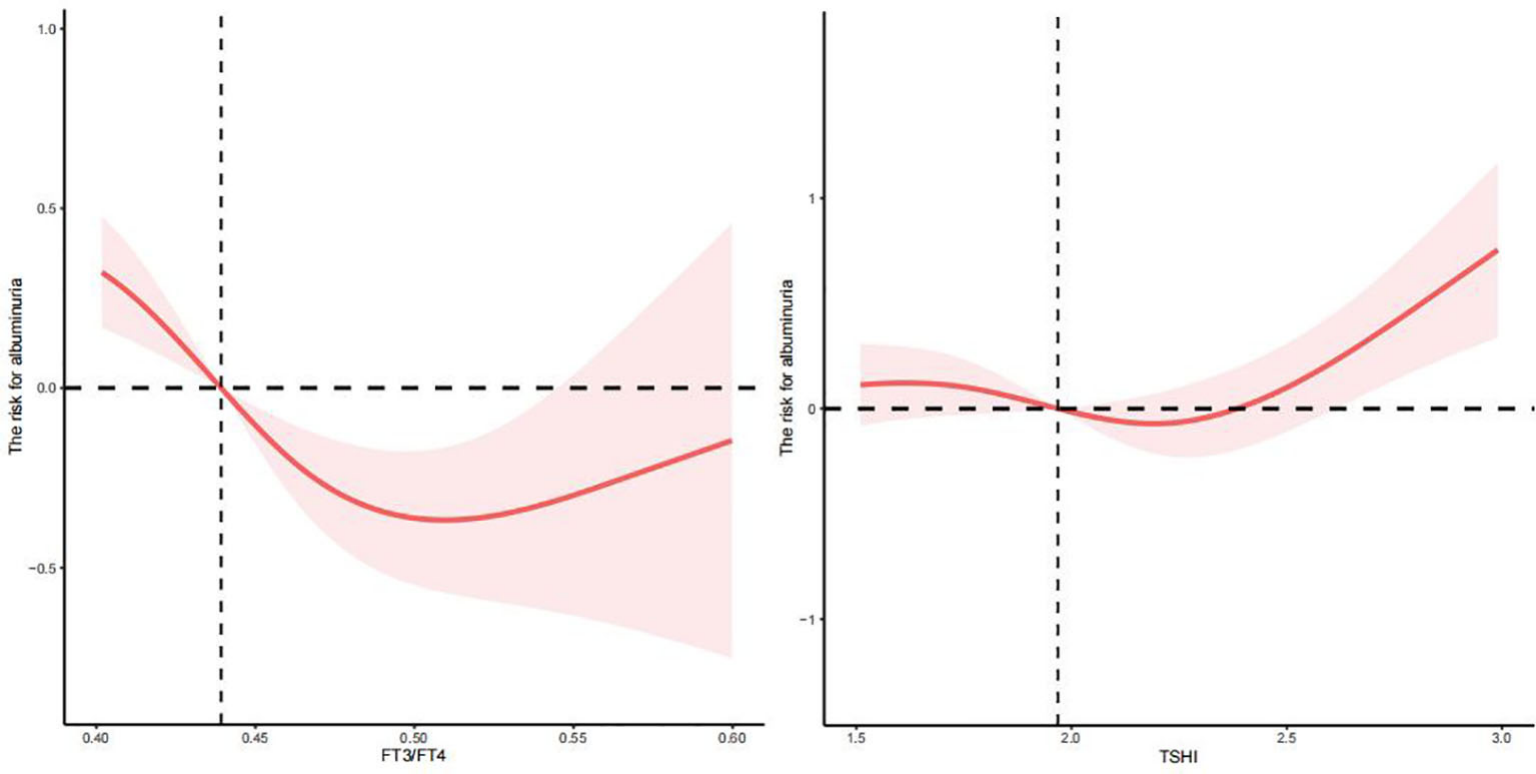
The nonlinear relationship between FT3/FT4, TSHI, and albuminuria.

There was a nonlinear relationship between FT3/FT4 and TSHI and albuminuria (*p* for all = 0, *p* for nonlinear = 0.0284; *p* for all = 0.0022, *p* for nonlinear =0.0113) (see **Figure 3**) but not TFQI_FT4_, TFQI_FT3_, TT4RI, and TT3RI (*p* for all >0.05 or *p* for nonlinear >0.05). After adjusting for age, sex, education level, race, smoking status, BMI, ALT, AST, triglycerides, total cholesterol, uric acid, eGFR, urine iodine, DM, and hypertension, we found that the relationship between FT3/FT4 and TSHI and albuminuria was approximately U-shaped. The changepoints between FT3/FT4 and TSHI with albuminuria were 0.510 and 2.188, respectively.

### 3.4 Subgroup analysis

**Figure 4:**
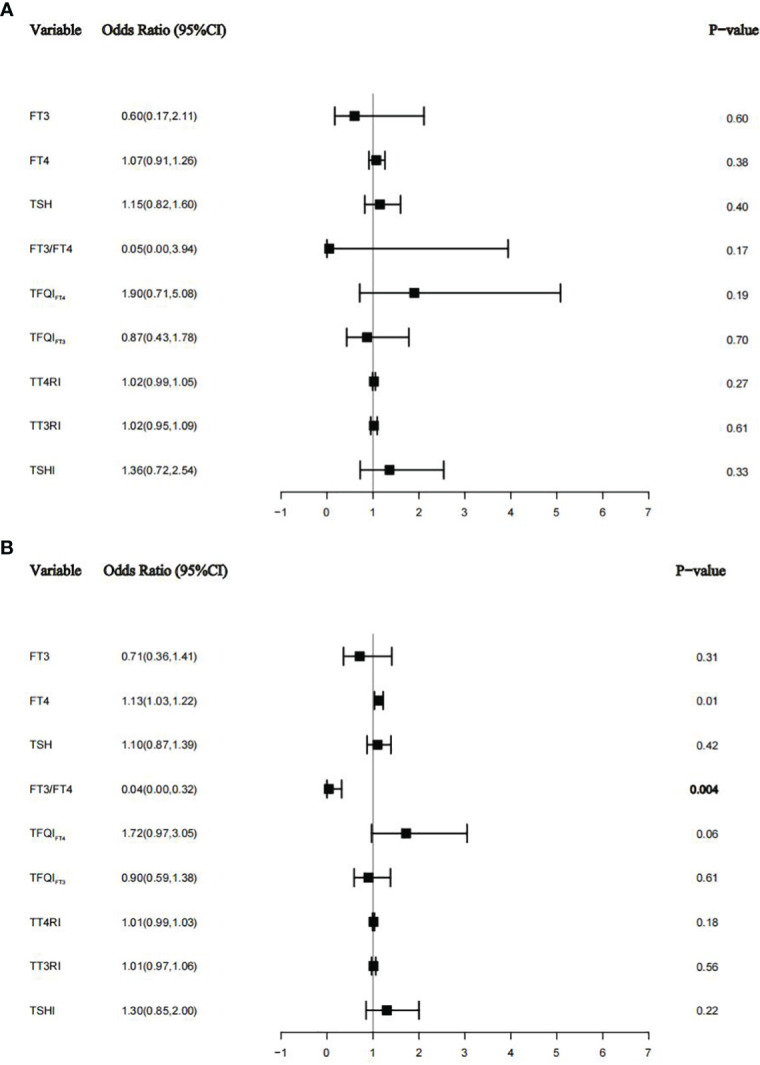
The relationship between thyroid function and thyroid homeostasis parameters with albuminuria in the diabetes group **(A)** and the hypertension group **(B)**.

Using subgroup analyses stratified by DM and hypertension, associations between thyroid function and thyroid homeostasis parameters and albuminuria were estimated. After adjusting for age, sex, education level, race, smoking status, BMI, ALT, AST, triglycerides, total cholesterol, uric acid, eGFR, urine iodine, DM, or hypertension, we found that there were similar results in hypertension populations (see **Figure 4B**) but not in diabetes populations (see **Figure 4A**). There was no significant difference between thyroid function and thyroid homeostasis parameters and albuminuria in the diabetes group (see **Figure 4A**), but there was a U-shaped nonlinear relationship between FT3 and albuminuria (*p* for all = 0.0005, *p* for nonlinear = 0.0003) (**Supplementary Figure 1**). However, in the hypertension group, FT4 was positively correlated with albuminuria (OR = 1.13; 95% CI = 1.03–1.22, *p* = 0.01), and FT3/FT4 was negatively correlated with albuminuria (OR = 0.04; 95% CI = 0.00–0.32, *p* = 0.004), which is basically consistent with our results.

### 3.5 ROC curves for optimal cutoff points of sensitivity to thyroid hormone indices

**Figure 5:**
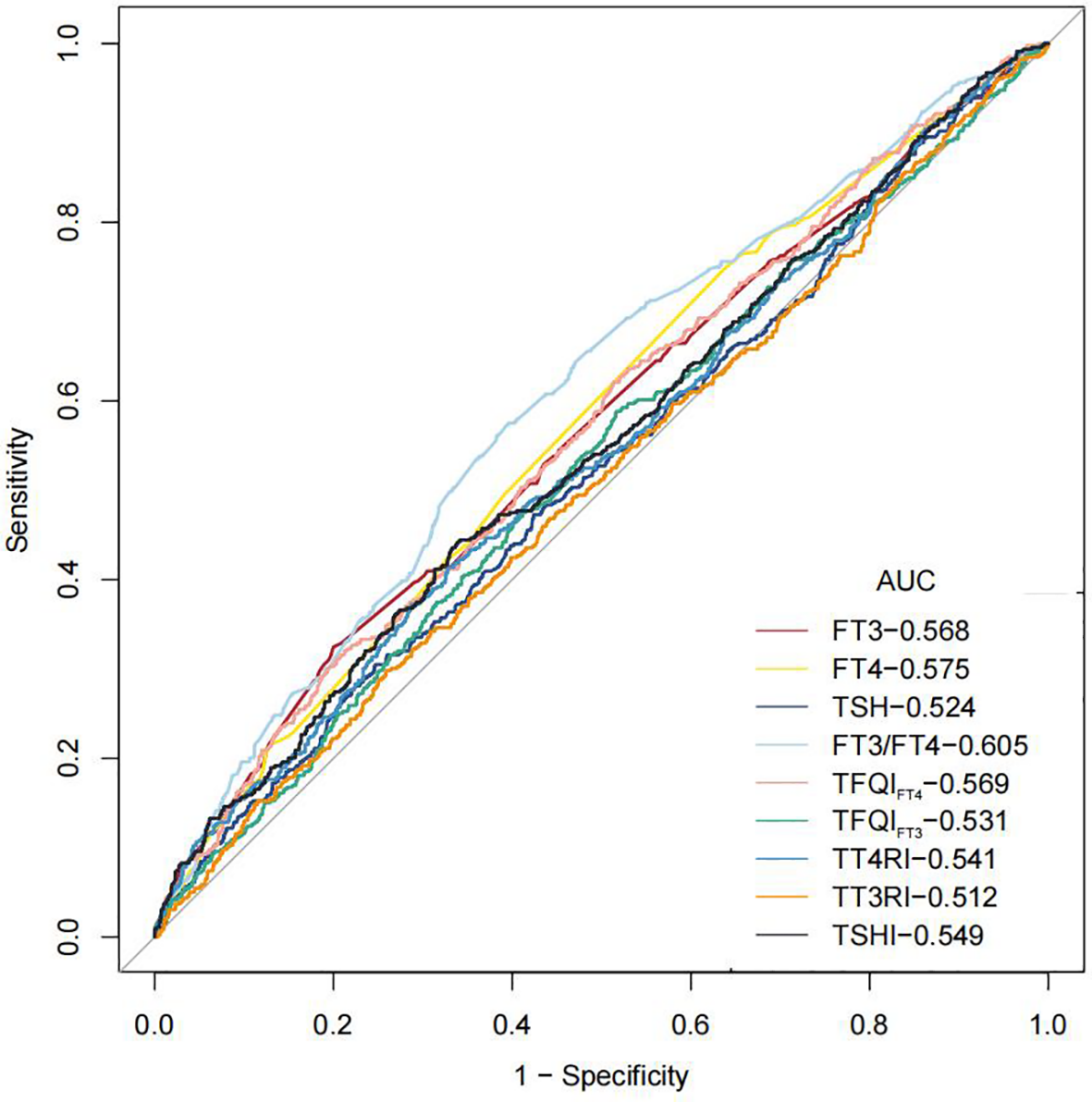
ROC curves for optimal cut-points of thyroid hormone and thyroid homeostasis parameters.

**Figure 5** shows that the areas under the curve (AUC) for FT3, FT4, TSH, FT3/FT4, TFQIFT4, TFQIFT3, TT4RI, TT3RI, and TSHI were 0.568 (95% CI 0.538–0.599), 0.575 (95% CI 0.545–0.604), 0.524 (95% CI 0.494–0.555), 0.605 (95% CI 0.575–0.634), 0.569 (95% CI 0.539–0.599), 0.531 (95% CI 0.501–0.562), 0.541 (95% CI 0.510–0.572), 0.512 (95% CI 0.481–0.543), and 0549 (95% CI 0.519–0.580), respectively. FT3/FT4 performed better than TFQI, TT4RI, and TSHI in ROC analyses for albuminuria prediction. The optimal cutoff point of FT3/FT4 for albuminuria prediction was 0.429, and the values of sensitivity and specificity of this cutoff point were 0.604 and 0.575, respectively.

## 4 Discussion

In our study, we found a relationship between FT4, FT3/FT4, TFQI_FT4_, and TSHI and albuminuria, while in the diabetes group, there was a U-shaped nonlinear relationship between FT3 and albuminuria. The results show that there was a strong relation between thyroid tests and albuminuria, which provides new evidence for the relationship between thyroid and renal function and provides a rationale for the evaluation of thyroid function in patients with albuminuria and people at high risk of albuminuria.

In recent years, there has been a more comprehensive assessment of the connection between thyroid dysfunction and kidney diseases (24–27). The kidneys not only serve as organs for the metabolism and elimination of thyroid hormones but also act as target organs for certain effects of iodine thyroid hormones (25). Our results showed that FT4 was positively correlated with albuminuria, possibly via one or both of the following mechanisms. The elevation of FT4 may be associated with glomerular hyperfiltration and high pressure, changes in tubular handling of proteins, or alterations in the structure of the glomerular barrier (28). FT4 may also lead to glomerular hyperfiltration (29). Thyroid hormones regulate endothelial function and homeostasis by dilating blood vessels and inhibiting angiotensin II receptors and their signal transduction; thus, they have an important relationship with endothelial injury and cardiovascular diseases (30–32). One hyperthyroidism rat model showed that an increase in FT4 can increase the level of ET-1 synthesis by endothelial cells in plasma, which can reflect the degree of injury to endothelial cells (33). At the same time, many studies have suggested that the increase in systemic vascular permeability is related to the increase in glomerular albumin leakage; that is, extensive endothelial injury precedes the production of microalbuminuria (34). Therefore, microalbuminuria is considered a comprehensive marker of systemic endothelial dysfunction and vascular disease (35). The association between FT4 and albuminuria by endothelial cell damage perhaps reflects one of the underlying mechanisms.

Our results showed that the relationship between FT3 and albuminuria was not statistically significant. There are two possible reasons for this: On the one hand, our study population was older people over the age of 60, whose T3 levels are lower than those of young and middle-aged people (36). On the other hand, albuminuria is usually one of the clinical manifestations of other chronic diseases or autoimmune diseases, such as diabetes (37), hypertension, and systemic lupus erythematosus (38). However, these primary diseases may themselves cause a decline in T3 levels. In subgroup analysis, our findings suggested that in the diabetes group, thyroid function affecting UAR is inconsistent, resulting in a U-shaped nonlinear relationship between FT3 and albuminuria in the diabetes group. A study showed that UACR levels were negatively associated with FT3 and T3 in patients with type 2 diabetes (14), and Shi et al. indicated that FT3 was negatively associated with UACR (39). What our studies have in common is that FT3 appears to be the most associated indicator of albuminuria in diabetic patients compared to FT4 and TSH. However, in our study, we were the first to explore the nonlinear relationship between FT3 and albuminuria, and we were surprised to find a U-shaped relationship between FT3 and albuminuria. There is nothing yet to claim a more accurate basis for thyroid management.

The secretion of thyroid hormone is controlled by the HPT axis. Thyrotropin-releasing hormone (TRH) from the hypothalamus stimulates the synthesis and release of TSH from the anterior pituitary. TSH is crucial throughout the stages of thyroid hormone production and secretion from the thyroid gland. Additionally, the levels of TRH and TSH are regulated by the negative feedback loop of thyroid hormones (2). Thyroid homeostasis parameters associated more with FT4 levels than with TSH levels. Individual indicators may not accurately describe thyroid status; in the evaluation and prediction of albuminuria, thyroid homeostasis parameters offer a more comprehensive assessment of thyroid status than individual thyroid indicators. Thyroid homeostasis parameters can be used to identify reduced sensitivity to thyroid hormone. The reduced sensitivity of thyroid hormones is closely associated with obesity, metabolic syndrome, and diabetes, even in the euthyroid population (5). The first measures used to evaluate central sensitivity were TT4RI and TSHI. Chen et al. demonstrated that elevated TSHI and TT4RI were associated with an increased prevalence of kidney disorders in type 2 diabetes patients (14). A higher value of TFQI_FT4_ indicated higher TSH than that expected for the actual FT4, indicating a lower sensitivity to FT4 (5). The diminished suppression of TSH not only may indicate a decreased sensitivity to thyroid hormones but also could signify an elevated set point of TSH and thyroid hormone, a characteristic often observed in type 2 allostasis (40). Allostasis, characterized by a dynamic response to maintain stability, comprises two categories of allostatic load. Prolonged exposure to type 2 allostatic load can contribute to conditions such as obesity, hypertension, type 2 diabetes, and dyslipidemia (40). Research has highlighted a significant correlation between metabolic disorders resulting from type 2 allostatic load and renal dysfunction (15). Our results showed that TFQI_FT4_ was positively correlated with albuminuria (OR = 1.91; 95% CI = 1.20–3.04, *p* = 0.01) in individuals over 60 years old, again proving the close relationship between type 2 allostatic load and renal dysfunction (15).

In a prospective observational cohort study involving a British population, it was revealed that a low FT3/FT4 ratio was linked to frailty and long-term mortality in older hospitalized patients (41), and many studies have also shown that FT3/FT4 was negatively correlated with disease mortality and adverse outcomes (42–44). A low FT3/FT4 ratio seemed to be a particularly negative indicator for older people, and our results also provided evidence for this. Our results show that FT3/FT4 was closely related to albuminuria in the elderly (OR = 0.06; 95% CI = 0.01–0.46, *p* = 0.01). FT3/FT4 performed better than other parameters on ROC analyses for albuminuria prediction and may be used as a helpful indicator to predict albuminuria and provide novel ideas for the evaluation and treatment of albuminuria.

Our study utilized nationwide data, incorporating sample weights, thereby enhancing the generalizability of our findings to the broader older population in the United States. Nevertheless, it is important to acknowledge certain limitations. First, despite adjusting for various potential covariates, numerous factors influence UAE, and the potential impact of other confounding variables cannot be entirely eliminated. Second, our study was a cross-sectional study and cannot unravel causality. Therefore, future cohort studies or Mendelian randomization studies are needed to further confirm causality. Last, patients in our study population were older than 60 years old, so the conclusions may not be applicable to young or middle-aged people.

## 5 Conclusion

In conclusion, our study found that FT4, TFQI_FT4_, and a low FT3/FT4 ratio were risk factors for albuminuria in euthyroid individuals over 60 years old and that FT3/FT4 may be used as a helpful indicator to predict albuminuria. In the diabetes subgroup, there was a U-shaped nonlinear relationship between FT3 and albuminuria. Our study provides further data regarding the associations between the thyroid status and renal disease. Further studies may provide clinically relevant information.

## Data availability statement

The original contributions presented in the study are included in the article/**Supplementary Material**. Further inquiries can be directed to the corresponding author.

## Ethics statement

The studies involving humans were approved by National Center for Health Statistics Institutional Review Board. The studies were conducted in accordance with the local legislation and institutional requirements. The participants provided their written informed consent to participate in this study.

## Author contributions

XL: Writing – original draft. YL: Data curation, Writing – original draft. YC: Data curation, Writing – original draft. YZ: Validation, Writing – original draft. LZ: Validation, Writing – original draft. HZ: Writing – review & editing.
