# Supplementary material for: Thyroid function and thyroid homeostasis parameters are associated with increased urinary albumin excretion in euthyroid individuals over 60 years old from NHANES
Source: Front Endocrinol (Lausanne). 2024 Jan 8;14:1285249. doi: 10.3389/fendo.2023.1285249 (PMC10800926; doi:10.3389/fendo.2023.1285249)
Supplement: Supplementary file 1 [file DataSheet_1.zip › Supplementary Materials/Supplementary Tables/Supplementary Table 1.docx]

Supplementary table 1：The description of missing data.

| Variables | Missing | Non-missing | Missing Proportion |
| --- | --- | --- | --- |
| Age | 0 | 1985 | 0% |
| Sex | 0 | 1985 | 0% |
| Race | 0 | 1985 | 0% |
| Education levels | 0 | 1985 | 0% |
| Smoking | 0 | 1985 | 0% |
| BMI | 33 | 1952 | 1.7% |
| ALT | 4 | 1981 | 0.2% |
| AST | 4 | 1981 | 0.2% |
| Uric acid | 3 | 1982 | 0.2% |
| Triglyceride | 1000 | 985 | 50.4% |
| Total cholesterol | 0 | 1985 | 0% |
| Urine iodine | 34 | 1951 | 1.7% |
| eGFR | 1 | 1984 | 0.1% |
| Diabetes | 0 | 2286 | 0% |
| Hypertension | 0 | 2286 | 0% |
