# Supplementary material for: Thyroid function and thyroid homeostasis parameters are associated with increased urinary albumin excretion in euthyroid individuals over 60 years old from NHANES
Source: Front Endocrinol (Lausanne). 2024 Jan 8;14:1285249. doi: 10.3389/fendo.2023.1285249 (PMC10800926; doi:10.3389/fendo.2023.1285249)
Supplement: Supplementary file 1 [file DataSheet_1.zip › Supplementary Materials/Supplementary Tables/Supplementary Table 2.docx]

Supplement Table 2: Sensitivity comparative analysis between Pre-imputation and Post-imputation.

| Variable | Pre-imputation | Post-imputation | P value |
| --- | --- | --- | --- |
| eGFR | 73.40±19.16 | 73.39±19.16 | 0.98 |
| BMI | 28.75±5.65 | 28.74±5.63 | 0.93 |
| ALT | 22.96±23.37 | 22.96±23.35 | 0.99 |
| Triglyceride | 1.53±1.04 | 1.50±0.97 | 0.42 |
| Urine iodine | 426.29±2447.05 | 455.69±2731.16 | 0.72 |
