# Supplementary material for: Thyroid function and thyroid homeostasis parameters are associated with increased urinary albumin excretion in euthyroid individuals over 60 years old from NHANES
Source: Front Endocrinol (Lausanne). 2024 Jan 8;14:1285249. doi: 10.3389/fendo.2023.1285249 (PMC10800926; doi:10.3389/fendo.2023.1285249)
Supplement: Supplementary file 1 [file DataSheet_1.zip › Supplementary Materials/Supplementary Tables/Supplementary Table 3.docx]

| Variable | OR (95%CI) | P-value |
| --- | --- | --- |
| FT3 (pg/mL) | 0.62(0.34,1.11) | 0.10 |
| Age (years) | 1.07(1.03,1.11) | <0.001* |
| Sex |  |  |
| Male | Ref | Ref |
| Female | 0.88(0.59,1.30) | 0.51 |
| Race |  |  |
| Mexican american | Ref | Ref |
| Non-hispanic black | 0.50(0.27,0.94) | 0.03* |
| Non-hispanic white | 0.45(0.26,0.80) | 0.01* |
| Other hispanic | 0.52(0.26,1.02) | 0.06 |
| Other race | 0.77(0.34,1.74) | 0.51 |
| Education levels |  |  |
| less than 9th grade | Ref | Ref |
| 9-11th grade | 1.07(0.60,1.90) | 0.81 |
| more than high school | 0.79(0.49,1.28) | 0.33 |
| Smoking |  |  |
| Never smoker | Ref | Ref |
| Former smoker | 1.31(0.83,2.06) | 0.24 |
| Current smoker | 2.26(1.26,4.08) | 0.01* |
| BMI (kg/m^2^) | 0.99(0.96,1.01) | 0.33 |
| ALT (U/L) | 1.00(0.99,1.02) | 0.83 |
| AST (U/L) | 1.00(0.97,1.03) | 0.92 |
| Uric acid (umol/L) | 1.00(1.00,1.00) | 0.77 |
| Triglyceride (mmol/L) | 1.15(1.00,1.31) | 0.04* |
| Total cholesterol (mmol/L) | 0.99(0.84,1.18) | 0.93 |
| Urine iodine (ug/L) | 1.00(1.00,1.00) | 0.87 |
| Diabetes or not |  |  |
| No | Ref | Ref |
| Yes | 2.44(1.77,3.37) | <0.0001* |
| Hypertension or not |  |  |
| No | Ref | Ref |
| Yes | 2.04(1.35,3.09) | <0.001* |

Supplementary Table 3 The multivariate logistic regression between FT3 with albuminuria.

Adjusted for age, sex, education level, race, smoke, BMI, ALT, AST, triglyceride, total cholesterol, uric acid, eGFR, urine iodine, DM and Hypertension.

FT3 triiodothyronine, BMI body mass index, ALT glutamic-pyruvic transaminase, AST glutamic oxaloacetic transaminase, eGFR estimated glomerular filtration rate

*p<0.05
