# Supplementary material for: Thyroid function and thyroid homeostasis parameters are associated with increased urinary albumin excretion in euthyroid individuals over 60 years old from NHANES
Source: Front Endocrinol (Lausanne). 2024 Jan 8;14:1285249. doi: 10.3389/fendo.2023.1285249 (PMC10800926; doi:10.3389/fendo.2023.1285249)
Supplement: Supplementary file 1 [file DataSheet_1.zip › Supplementary Materials/Supplementary Tables/Supplementary Table 6.docx]

| Variable | OR (95%CI) | P-value |
| --- | --- | --- |
| FT3/FT4 | 0.03(0.00,0.26) | 0.003* |
| Age (years) | 1.07(1.03,1.11) | <0.001* |
| Sex |  |  |
| Male | Ref | Ref |
| Female | 0.87(0.59,1.30) | 0.49 |
| Race |  |  |
| Mexican american | Ref | Ref |
| Non-hispanic black | 0.53(0.28,1.00) | 0.05 |
| Non-hispanic white | 0.47(0.26,0.84) | 0.01* |
| Other hispanic | 0.52(0.27,1.03) | 0.06 |
| Other race | 0.75(0.33,1.71) | 0.48 |
| Education levels |  |  |
| less than 9th grade | Ref | Ref |
| 9-11th grade | 1.03(0.58,1.81) | 0.92 |
| more than high school | 0.77(0.48,1.24) | 0.27 |
| Smoking |  |  |
| Never smoker | Ref | Ref |
| Former smoker | 1.31(0.83,2.07) | 0.23 |
| Current smoker | 2.29(1.29,4.07) | 0.01* |
| BMI (kg/m^2^) | 0.99(0.96,1.01) | 0.37 |
| ALT (U/L) | 1.00(0.99,1.02) | 0.83 |
| AST (U/L) | 1.00(0.97,1.03) | 0.94 |
| Uric acid (umol/L) | 1.00(1.00,1.00) | 0.65 |
| Triglyceride (mmol/L) | 1.19(1.03,1.36) | 0.02* |
| Total cholesterol (mmol/L) | 1.00(0.84,1.18) | 0.96 |
| Urine iodine (ug/L) | 1.00(1.00,1.00) | 0.89 |
| Diabetes or not |  |  |
| No | Ref | Ref |
| Yes | 2.37(1.71,3.27) | <0.001* |
| Hypertension or not |  |  |
| No | Ref | Ref |
| Yes | 2.04(1.35,3.09) | 0.001* |

Supplementary Table 6 The multivariate logistic regression between FT3/FT4 with albuminuria.

Adjusted for age, sex, education level, race, smoke, BMI, ALT, AST, triglyceride, total cholesterol, uric acid, eGFR, urine iodine, DM and Hypertension.

FT3/FT4 FT3/FT4 ratio BMI body mass index, ALT glutamic-pyruvic transaminase, AST glutamic oxaloacetic transaminase, eGFR estimated glomerular filtration rate

*p<0.05
