# Supplementary material for: Thyroid function and thyroid homeostasis parameters are associated with increased urinary albumin excretion in euthyroid individuals over 60 years old from NHANES
Source: Front Endocrinol (Lausanne). 2024 Jan 8;14:1285249. doi: 10.3389/fendo.2023.1285249 (PMC10800926; doi:10.3389/fendo.2023.1285249)
Supplement: Supplementary file 1 [file DataSheet_1.zip › Supplementary Materials/Supplementary Tables/Supplementary Table 7.docx]

| Variable | OR (95%CI) | P-value |
| --- | --- | --- |
| TFQI_FT4_ | 1.79(1.08,2.99) | 0.03* |
| Age (years) | 1.07(1.03,1.11) | <0.001* |
| Sex |  |  |
| Male | Ref | Ref |
| Female | 0.90(0.60,1.34) | 0.58 |
| Race |  |  |
| Mexican american | Ref | Ref |
| Non-hispanic black | 0.58(0.30,1.12) | 0.10 |
| Non-hispanic white | 0.47(0.26,0.83) | 0.01* |
| Other hispanic | 0.52(0.26,1.04) | 0.06 |
| Other race | 0.79(0.35,1.83) | 0.57 |
| Education levels |  |  |
| less than 9th grade | Ref | Ref |
| 9-11th grade | 1.06(0.60,1.86) | 0.83 |
| more than high school | 0.78(0.48,1.25) | 0.28 |
| Smoking |  |  |
| Never smoker | Ref | Ref |
| Former smoker | 1.32(0.83,2.09) | 0.23 |
| Current smoker | 2.33(1.28,4.24) | 0.01* |
| BMI (kg/m^2^) | 0.98(0.96,1.01) | 0.25 |
| ALT (U/L) | 1.00(0.98,1.02) | 0.93 |
| AST (U/L) | 1.00(0.97,1.03) | 0.99 |
| Uric acid (umol/L) | 1.00(1.00,1.00) | 0.75 |
| Triglyceride (mmol/L) | 1.12(0.98,1.29) | 0.09 |
| Total cholesterol (mmol/L) | 1.00(0.84,1.18) | 0.96 |
| Urine iodine (ug/L) | 1.00(1.00,1.00) | 0.77 |
| Diabetes or not |  |  |
| No | Ref | Ref |
| Yes | 2.47(1.76,3.45) | <0.0001* |
| Hypertension or not |  |  |
| No | Ref | Ref |
| Yes | 2.00(1.32,3.04) | 0.002* |

Supplementary Table 7 The multivariate logistic regression between TFQI_FT4_ with albuminuria.

Adjusted for age, sex, education level, race, smoke, BMI, ALT, AST, triglyceride, total cholesterol, uric acid, eGFR, urine iodine, DM and Hypertension.

TFQI_FT4_ thyroid Feedback Quantile-based Index, BMI body mass index, ALT glutamic-pyruvic transaminase, AST glutamic oxaloacetic transaminase, eGFR estimated glomerular filtration rate

*p<0.05
