# Supplementary material for: Thyroid function and thyroid homeostasis parameters are associated with increased urinary albumin excretion in euthyroid individuals over 60 years old from NHANES
Source: Front Endocrinol (Lausanne). 2024 Jan 8;14:1285249. doi: 10.3389/fendo.2023.1285249 (PMC10800926; doi:10.3389/fendo.2023.1285249)
Supplement: Supplementary file 1 [file DataSheet_1.zip › Supplementary Materials/Supplementary Tables/Supplementary Table 8.docx]

| Variable | OR (95%CI) | P-value |
| --- | --- | --- |
| TFQI_FT3_ | 0.84(0.54,1.31) | 0.42 |
| Age (years) | 1.07(1.04,1.11) | <0.001* |
| Sex |  |  |
| Male | Ref | Ref |
| Female | 0.88(0.60,1.30) | 0.51 |
| Race |  |  |
| Mexican american | Ref | Ref |
| Non-hispanic black | 0.50(0.27,0.95) | 0.04* |
| Non-hispanic white | 0.47(0.27,0.82) | 0.01* |
| Other hispanic | 0.51(0.26,1.02) | 0.06 |
| Other race | 0.76(0.33,1.75) | 0.50 |
| Education levels |  |  |
| less than 9th grade | Ref | Ref |
| 9-11th grade | 1.07(0.60,1.91) | 0.82 |
| more than high school | 0.79(0.49,1.27) | 0.32 |
| Smoking |  |  |
| Never smoker | Ref | Ref |
| Former smoker | 1.30(0.83,2.05) | 0.24 |
| Current smoker | 2.22(1.23,4.02) | 0.01* |
| BMI (kg/m^2^) | 0.99(0.96,1.01) | 0.31 |
| ALT (U/L) | 1.00(0.99,1.02) | 0.90 |
| AST (U/L) | 1.00(0.97,1.03) | 1.00 |
| Uric acid (umol/L) | 1.00(1.00,1.00) | 0.70 |
| Triglyceride (mmol/L) | 1.14(1.00,1.30) | 0.05 |
| Total cholesterol (mmol/L) | 0.99(0.83,1.16) | 0.86 |
| Urine iodine (ug/L) | 1.00(1.00,1.00) | 0.76 |
| Diabetes or not |  |  |
| No | Ref | Ref |
| Yes | 2.46(1.78,3.41) | <0.0001* |
| Hypertension or not |  |  |
| No | Ref | Ref |
| Yes | 2.03(1.34,3.07) | 0.002* |

Supplementary Table 8 The multivariate logistic regression between TFQI_FT3_ with albuminuria.

Adjusted for age, sex, education level, race, smoke, BMI, ALT, AST, triglyceride, total cholesterol, uric acid, eGFR, urine iodine, DM and Hypertension.

TFQI_FT3_ thyroid Feedback Quantile-based Index, BMI body mass index, ALT glutamic-pyruvic transaminase, AST glutamic oxaloacetic transaminase, eGFR estimated glomerular filtration rate

*p<0.05
