# Supplementary figures and images for: Thyroid function and thyroid homeostasis parameters are associated with increased urinary albumin excretion in euthyroid individuals over 60 years old from NHANES
Source: Front Endocrinol (Lausanne). 2024 Jan 8;14:1285249. doi: 10.3389/fendo.2023.1285249 (PMC10800926; doi:10.3389/fendo.2023.1285249)

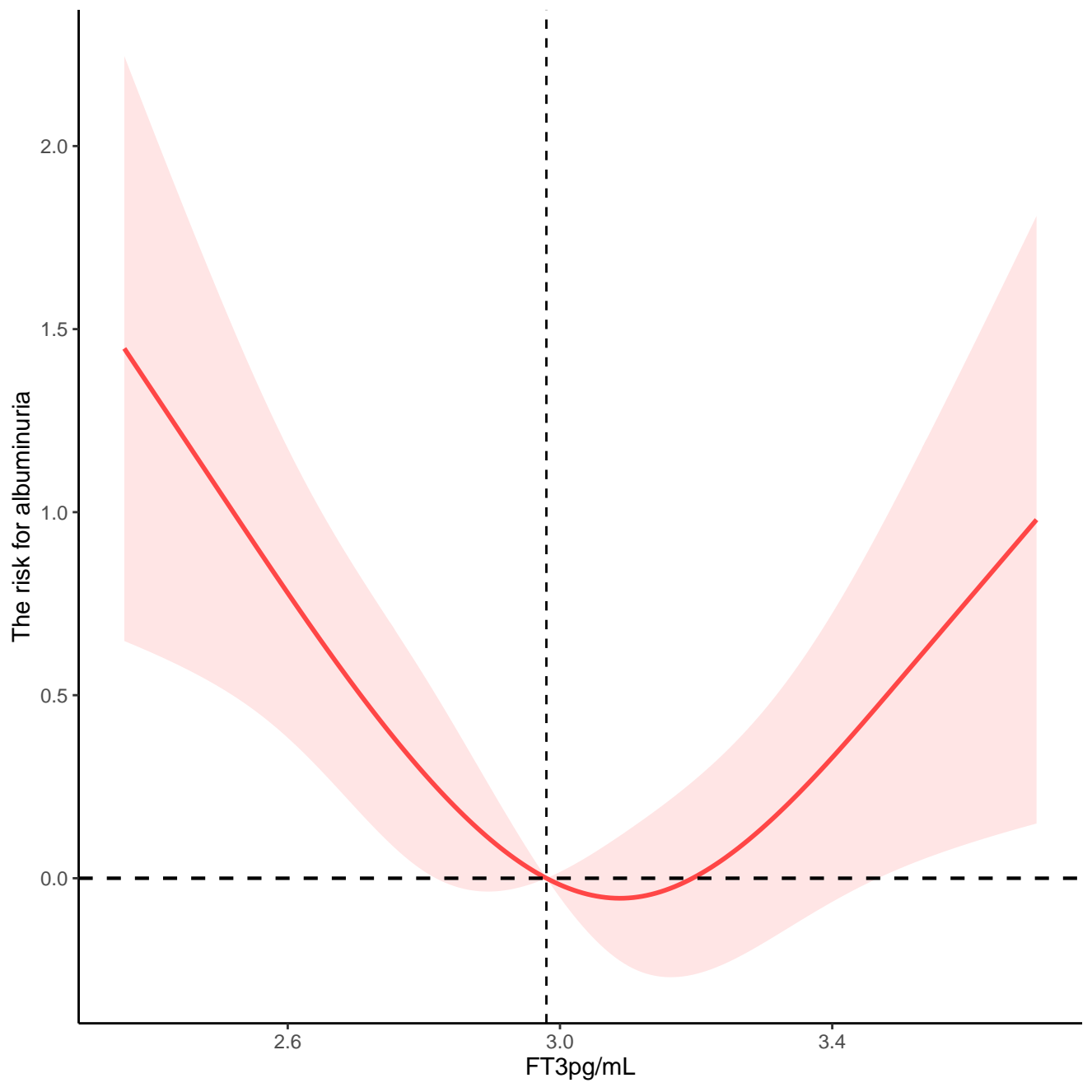

Supplement: Supplementary file 1 [file DataSheet_1.zip › Supplementary Materials/Supplementary Figures/Supplementary Figure 1.pdf]
